# The effects of different types of exercise on cognitive function in the older adults: a systematic review and network meta-analysis

**DOI:** 10.3389/fpubh.2026.1802431

**Published:** 2026-06-23

**Authors:** Hailong Jiao, Yong He, Yuxuan Ying, Meng Tao, Tianqi Xia, Xianyou Cui

**Affiliations:** 1 School of Physical Education, Zhejiang Guangsha Vocational and Technical University of Construction, Dongyang, China; 2 School of Exercise and Health, Shanghai University of Sport, Shanghai, China; 3 School of Physical Education, Shihezi University, Xinjiang, China; 4 Modern Service College, HeFei College of Finance and Economics, Hefei, China

**Keywords:** aerobic training, cognitive function, older adults people, multiple combination training, resistance training

## Abstract

**Objective:**

Global population aging is accelerating, and age-related cognitive decline has emerged as a core public health concern impacting the health and quality of life of older adults. Physical exercise, a low-cost, low-risk non-pharmacological intervention, has been validated to delay cognitive decline. However, there is no consensus yet on the relative effectiveness of different exercise types in improving cognitive function among older adults individuals with cognitive health issues and those with MCI. This highlights the urgent need to determine the best intervention strategies through evidence-based medicine.

**Methods:**

A systematic literature search was conducted across PubMed, the Cochrane Library, Embase, SCOPUS, Web of Science and CNKI, with a search cutoff date of December 31, 2025. Initial database searches and reference list screening identified 3,816 records. Effect sizes were synthesized using the standardized mean difference (SMD) and 95% confidence intervals (CIs). The intervention efficacy of different exercise types was ranked via Surface Under the Cumulative Ranking Curve (SUCRA) values. Subgroup analyses, sensitivity analyses, and publication bias assessments were also performed.Statistical analyses adopted a random-effects consistency model for network Meta-analysis, with node-splitting method to test inconsistency.

**Results:**

A total of 18 randomized controlled trials (RCTs) involving 1,316 older adults were ultimately included. Meta-analysis results demonstrated that various exercise types exerted significant beneficial effects on cognitive function in older adults: MMSE, MT yielded the optimal intervention effect; MoCA, ST and RT were the most effective; WAIS, ST and AT demonstrated significant benefits; WCST, AT exhibited a statistically significant intervention effect (SMD = 0.61, 95% CI: 0.11–1.12).

**Conclusion:**

Physical exercise effectively enhances multi-dimensional cognitive function in older adults, with distinct intervention advantages associated with different exercise types. In clinical practice, MT, RT, ST, or AT may be prioritized as intervention strategies based on the individual's cognitive assessment results and health status. It is recommended to adopt a moderate-intensity exercise regimen of “60–90 min per session, ≥3 times per week, and lasting ≥12 weeks” to maximize cognitive benefits. This study provides high-level evidence-based medical support for formulating exercise prescriptions to maintain cognitive function in older adults.

## Introduction

Population aging has emerged as a core concern in global public health. According to the United Nations' World Population Prospects report, the global population is projected to reach 9.8 billion by 2050, with over 1.5 billion individuals aged 60 years and above (accounting for 16% of the total population). Data from China's seventh national census indicate that the number of people aged 60 years and above in China has reached 190 million (13.5% of the total population), marking the country's official entry into an aging society.

Aging is often accompanied by declines in physical function and cognitive ability. Cognitive function encompasses memory, language, visuospatial skills, and executive function, among other domains (1). Most individuals aged 60 years and above experience varying degrees of cognitive decline, which not only reduces quality of life and impairs self-care capacity but also may shorten life expectancy and increase mortality risk (2). Clarifying the mechanisms underlying cognitive aging and exploring effective preventive strategies have therefore become priority research directions in clinical and public health fields.

Among potential preventive interventions, physical exercise can enhance cognitive function by regulating neural and vascular adaptive mechanisms—such as promoting neurogenesis, angiogenesis, reducing inflammatory responses, and mitigating oxidative stress damage (3). As a non-pharmacological therapy, physical exercise is widely recognized for its low cost, low risk, and accessibility. It serves not only as a key strategy for preventing non-communicable diseases but also improves mental health and maintains overall health status. Furthermore, accumulating evidence from multiple studies confirms that physical exercise can improve cognitive function in older adults and slow age-related cognitive decline (4, 5). However, most existing research focuses on the general effects of exercise, with no clear consensus regarding the specific associations between exercise and cognitive improvement in healthy older adults (6). Over the past few decades, numerous randomized controlled trials (RCTs) have explored the impact of exercise on cognition in older adults (7, 8), but findings remain inconsistent and lack uniformity.

MMSE is a widely used global cognitive assessment tool that can quickly reflect an individual's cognitive level and the severity of cognitive impairment (9). Nevertheless, a single scale is insufficient to cover the full spectrum of cognitive domains, necessitating a combination of multiple assessment methods. Based on these gaps, the present study aims to address two core research questions: (1) What are the intervention effects of different exercise types on the cognitive abilities of older adults? (2) What is the specific impact of exercise on the results of distinct cognitive tasks in older adults?

## Methods

### Search strategy

A comprehensive systematic search was conducted across PubMed, the Cochrane Library, Embase, SCOPUS, Web of Science, CNKI, Wanfang Data, and the VIP Chinese Science and Technology Periodical Database, encompassing all relevant articles published up to December 31, 2025. The key search terms used in this study included: “cognition,” “cognitive ability,” “cognitive decline,” “memory,” “older adults,” and “exercise.” To ensure methodological rigor and transparency, the study protocol was registered and approved on the PROSPERO international prospective register of systematic reviews prior to commencement (registration number: CRD420251166462). The detailed search strategy for each database is presented in Table 1.

**Table 1:** Search criteria in PubMed database.

Step	Search expression
#1	(((((((((“Exercise”[Mesh]) OR (“sport^*^”[Title/Abstract])) OR (“physical exercise”[Title/Abstract])) OR (“exercise intervention”[Title/Abstract])) OR (“aerobic training”[Title/Abstract])) OR (“resistance training”[Title/Abstract])) OR (“strength training”[Title/Abstract])) OR (“stretching training”[Title/Abstract])) OR (“multicomponent exercise”[Title/Abstract])) OR (“mixed training”[Title/Abstract])
#2	(((“Aged”[Mesh])) OR (“older adults^*^”[Title/Abstract]) OR (“old people”[Title/Abstract])) OR (“older adults people”[Title/Abstract])
#3	(((“Cognitive”[Mesh]) OR (“cognitive ability[Title/Abstract])) OR (“cognitive function”[Title/Abstract])) OR (“cognitive decline”[Title/Abstract])
#4	#1 AND #2 AND #3

### Inclusion and exclusion criteria

Inclusion Criteria: (i) Study participants were aged 60 years or older (consistent with the WHO's definition of the older adults population for developing countries and China's demographic research standards); (ii) Participants were either cognitively healthy older adults or individuals with MCI; (iii) The core intervention was structured physical exercise with clear operational definitions: minimum 30 min per session, at least 1 time weekly, moderate intensity, and supervised or standardized in protocol. Multicomponent interventions were eligible only if physical exercise was the main component; interventions with cognitive training, lifestyle guidance, or education as the dominant content were excluded; (iv) The control group received non-exercise control conditions, including routine home care, general health education without structured physical activity, or maintaining usual lifestyle. All control conditions were defined as “no structured exercise intervention” to ensure a consistent comparison core; (v) Study outcomes included cognitive ability assessment using at least one standardized neuropsychological tool; (vi) The study design was limited to randomized controlled trials (RCTs).

Exclusion Criteria: (i) Studies failing to meet any of the above inclusion criteria; (ii) Studies with no available data for statistical analysis; (iii) Non-clinical trial study types, including reviews, basic research, observational studies, dissertations, case reports, and conference abstracts/reports; (iv) The research design was a non-randomized controlled trial; (v) Studies enrolling patients with confirmed dementia, stroke-related severe cognitive impairment, or other progressive neurological diseases.

### Study selection and data extraction

Two independent researchers (TM and YXY) screened studies in accordance with the predefined inclusion and exclusion criteria. Disagreements between the two researchers were resolved through discussion with a third researcher (JMF) or consultation with relevant experts.

The following data were extracted from the included studies: (i) Publication details: author(s) and publication year; (ii) Study characteristics: sample size and intervention type; (iii) Participant characteristics: age and baseline cognitive function; (iv) Outcome measures and Quantitative data for each group: number of participants, mean values, and standard deviations (SD) before and after the intervention.

Notably, exercise intensity, intervention adherence, and supervision status were not consistently reported across the included studies; therefore, these variables could not be systematically extracted or quantitatively analyzed.

### Quality assessment

Two independent researchers used the Cochrane Risk of Bias Tool to assess the risk of bias in included studies across seven domains: random sequence generation, allocation concealment, participant blinding, assessor blinding, data completeness, selective outcome reporting, and other potential sources of bias. Each domain was categorized into one of three risk levels: low risk of bias, unclear risk of bias, or high risk of bias. Disagreements between the two researchers were resolved through discussion with a third independent researcher or consultation with relevant experts.

### Outcome indicator

In the present study, a meta-analysis was conducted to synthesize the results of included studies, focusing on outcomes measured using the same cognitive assessment indicators. The primary cognitive ability assessment tools included were: MMSE, MoCA, WAIS, and WCST.

### Statistical analysis

Statistical analysis was performed using Review Manager 5.4 software and Stata 18.0 software. All outcome indicators were continuous variables. For outcomes measured using the same method and units, the MD and 95% CI were used as effect size indicators. For outcomes with different measurement methods or units, the SMD and 95% CI were used instead. The Cochrane Risk of Bias Assessment Tool was used to evaluate the quality of included RCTs, covering six dimensions: random sequence generation, allocation concealment, blinding (participant/researcher blinding), data completeness, selective reporting, and other biases. The leave-one-out method was used to assess the robustness of the meta-analysis results. By sequentially excluding each included study and recalculating the combined effect size, the impact of a single study on the overall result was observed. If the direction and statistical significance of the combined effect size did not change significantly, the result was considered robust. Stata 18.0 software was used for league table analysis, network relationship analysis, and SUCRA ranking to compare the effects of different oxygen concentration groups on each outcome indicator. Publication bias was assessed using funnel plots (visual symmetry inspection) and Egger's regression test (quantitative assessment). A *P* > 0.05 was considered indicative of no statistically significant publication bias.

To verify the transitivity assumption of network meta-analysis, we evaluated the comparability of potential effect modifiers (including baseline cognitive status, intervention intensity, intervention duration, and participant age) across different intervention groups. Specifically, we presented descriptive baseline characteristics for each intervention group and performed statistical comparisons: one-way ANOVA was applied for continuous variables (age, baseline cognitive scores), and chi-square test was used for categorical variables (cognitive health status, gender). No significant between-group differences were observed for key modifiers (all P > 0.05), indicating that the transitivity assumption was adequately satisfied. Additionally, we performed a node-splitting analysis to detect local inconsistency, with no significant inconsistency (*P* > 0.05) indicating that the transitivity assumption was satisfied.

### Certainty of the evidence

Evidence of effectiveness for each study was combined with quality scores for use in discussing the results. The Grading of Recommendations Assessment, Development, and Evaluation (GRADE) methodology was used to rate the certainty of the evidence as “high,” “moderate,” “low,” or “very low.” GRADE was completed by two researchers, with differences resolved through consensus. This comprehensive assessment rates evidence as follows: (1) the risk of bias, downgraded by one level if “some concerns” and two levels if “high risk” of bias; (2) inconsistency, downgraded by one level when the impact of statistical heterogeneity (P) is moderate (>25%) and by two levels when high >75%; (3) imprecision: downgraded by one level when statistical power < 80% and if there was no clear direction of the effects; (4)risk of publication bias: downgrade one level if Egger's test < 0.05.

## Results

### Search results

**Figure 1:**
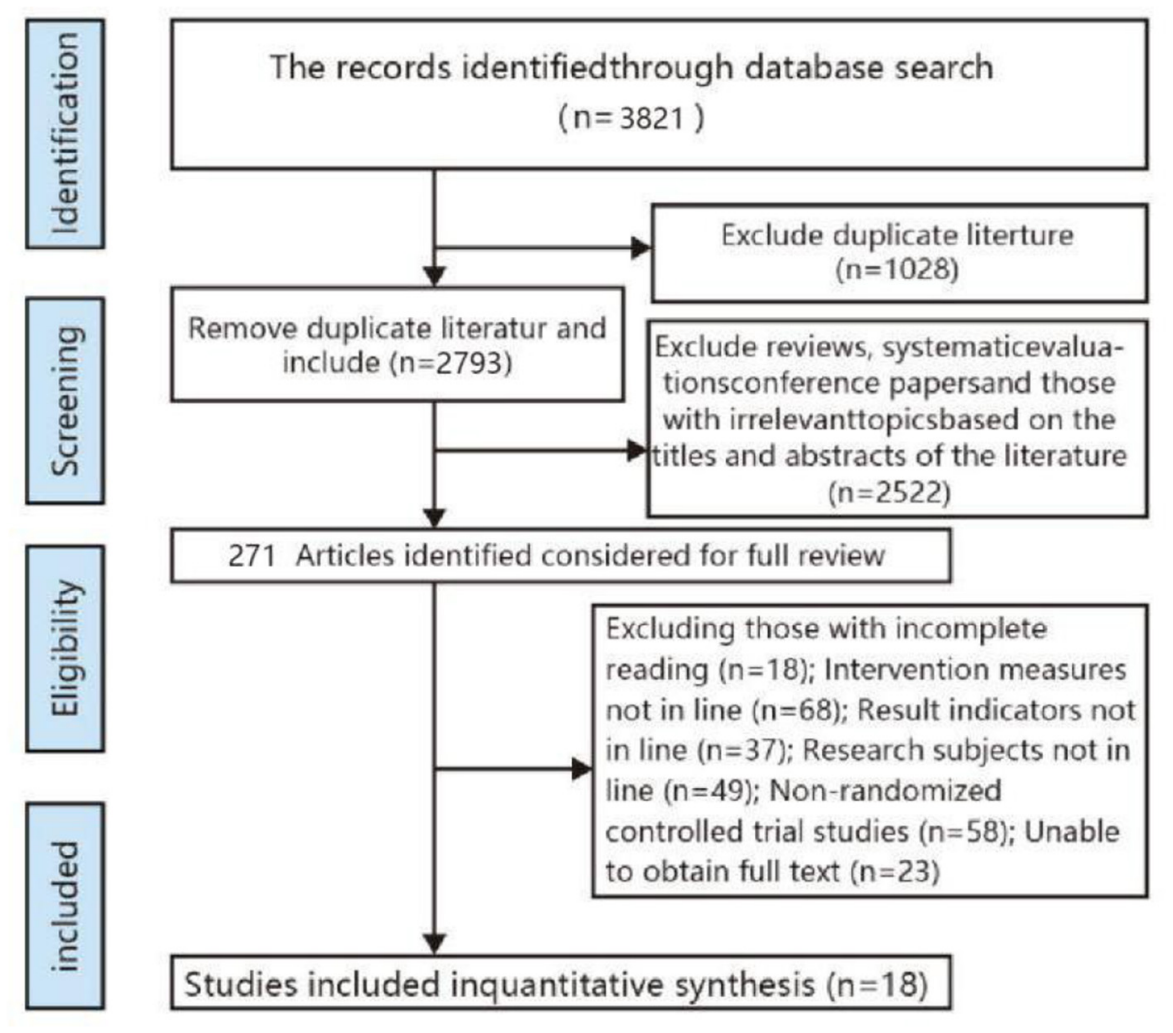
Flow diagram of the study selection.

A total of 3,821 articles were initially retrieved. After screening titles and abstracts, 1,028 duplicate articles and 2,522 irrelevant studies were excluded. Subsequent full-text assessment led to the exclusion of an additional 253 studies that failed to meet the inclusion criteria, including 58 non-randomized controlled trials, 18 duplicate reports, 23 studies with unavailable full texts, 49 studies with ineligible participant populations, 68 studies with inconsistent intervention measures, and 37 studies with inconsistent outcome indicators. Finally, 18 articles were included in the analysis (shown in Figure 1) (7, 8, 10–25). As of the study initiation, the characteristics of the included studies are presented in Table 2.

**Table 2:** Characteristics of 18 included studies.

Study	Sample size (IG/CG)	Age (IG/CG)	IG vs. CG	Session duration	Duration days (D/W)	Outcome
Cassilhas et al. (10)	23/39	68.40 ± 0.67	RT vs. Non	60 min	3/24	WAIS
Albinet et al. (8)	12/12	70.90 ± 4.90	AT vs. ST	60 min	3/12	MMSE;WCST
Kimura et al. (11)	65/54	73.60 ± 4.70	RT vs. Ed	90 min	2/12	MMSE
Muscari et al. (12)	60/60	68.80 ± 2.50	AT vs. Non	60 min	3/52	MMSE
Mortimer et al. (13)	30/30	67.30 ± 5.30	AT vs. Non	50 min	3/40	WAIS
Voss et al. (14)	35/35	55.00–80.00	RT vs. Non	40 min	3/52	MMSE
Fiatarone et al. (15)	22/27	aged 55 or above	RT vs. Non	75 min	3/26	WAIS
Fiatarone et al. (15)	22/27	aged 55 or above	CT vs. Non	75 min	3/26	WAIS
Ansai et al. (16)	23/23	82.80 ± 2.80	RT vs. Non	60 min	1/16	MoCA
Ansai et al. (16)	23/23	81.90 ± 1.90	MT vs. Non	60 min	1/16	MoCA
Antunes et al. (17)	23/17	64.04 ± 3.19	AT vs. Non	60 min	3/26	MMSE;WAIS;WCST
Tsai et al. (18)	24/24	70.79 ± 3.39	RT vs. Non	60 min	3/48	MMSE
Albinet et al. (7)	19/17	67.00 ± 5.00	AT vs. Non	60 min	2/21	MMSE
Yoon et al. (19)	19/19	75.00 ± 3.46	RT vs. Non	60 min	2/12	MMSE;MoCA
Song et al. (20)	60/60	76.22 ± 5.76	AT vs. Ed	60 min	3/16	MoCA
Li et al. (21)	45/45	60 years old or over	MT vs. Non	60 min	1/26	MMSE;MoCA
Chen et al. (22)	110/111	67.56 ± 4.99	AT vs. Non	30 min	3/24	MoCA
Huang et al. (23)	10/10	63.80 ± 7.50	AT vs. Non	120 min	5/2	MMSE;MoCA
Kwan et al. (24)	146/147	75.20 ± 7.10	AT vs. Non	60 min	2/8	MoCA
Wang et al. (25)	59/57	66.39 ± 4.24	MT vs. Ed	30 min	3/28	MMSE

IG stands for Intervention group; CG, Control group; AT, Aerobic training; RT, Resistance training; MT, Mixed training; CT, Cognitive training; ST, Stretching training; Ed, Education.

### Study characteristics

In this comprehensive analysis, 18 randomized controlled trial (RCT) articles were included (7, 8, 10–25). All included studies were published by the year 2025 and involved a total of 1,316 participants. Most studies enrolled cognitively healthy older adults, and several included individuals with MCI; no studies included patients with diagnosed dementia or severe neurological disorders. Control groups across studies were unified as non-exercise controls, including routine care, health education, or usual lifestyle. All controls lacked structured physical exercise, ensuring a consistent basis for comparison.

Most of the studies included mixed-gender participant groups. Regarding exercise types, all 18 trials focused on physical exercise as the intervention. The duration of exercise interventions ranged from 2 to 52 weeks, with each study employing its own unique intervention duration and frequency. Exercise intensity was not consistently reported across studies. Detailed characteristics of the included studies are presented in Table 2.

### Quality assessment of the included studies

**Figure 2:**
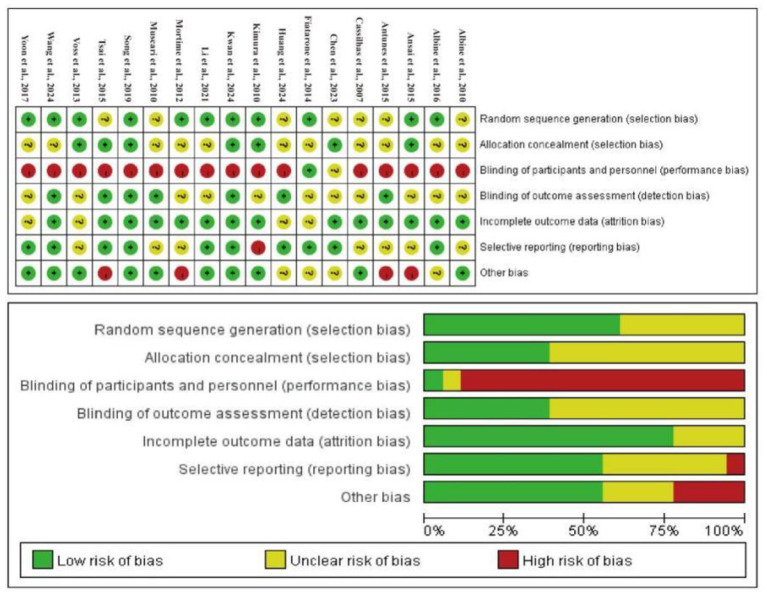
Risk assessment diagram for bias in the included studies.

Among the 18 included studies (7, 8, 10–25), 11 reported random sequence generation, and 7 reported allocation concealment measures. Owing to the inherent nature of exercise interventions (which are difficult to blind), all but 2 studies were judged to have a high risk of bias in participant and personnel blinding. Seven studies described the implementation of outcome assessor blinding, and all included studies had complete data. One study was assessed as having a high risk of selective reporting bias, as its reported outcome measures were inconsistent with those specified in the study protocol. Four studies provided no information on other potential sources of bias. A summary of the risk of bias assessment for all included studies is presented in Figure 2.

### The results of the meta-analysis

Transitivity assumption evaluation confirmed that key effect modifiers (baseline cognitive status, intervention intensity, duration, and participant age) were comparable across intervention groups (all *P* > 0.05 for between-group differences). Node-splitting analysis for local inconsistency also revealed no significant inconsistency (*P* > 0.05 for all closed loops), supporting the validity of the transitivity assumption and the use of the consistency model for network meta-analysis.

#### MMSE

This study quantitatively evaluated the regulatory effects of different exercise interventions on cognitive function in older adults via network meta-analysis. A total of 11 RCTs involving 721 participants were included, with 371 in the intervention group and 350 in the control group. In the network plot, node size represented the number of studies investigating each intervention, while the thickness of connecting lines indicated the frequency of direct comparisons between intervention pairs.

**Figure 3:**
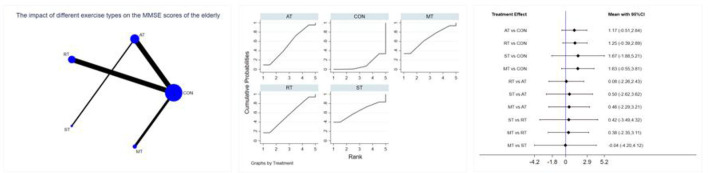
The network relationship diagram of different exercise intervention on MMSE.

Results showed that direct comparison studies between the control group (CON), AT and RT were the most abundant (reflected by the thickest connecting lines). The inconsistency test revealed no statistically significant inconsistency (*P* = 0.75), supporting the use of a consistency model for evidence synthesis. Both local inconsistency (node-splitting) and global inconsistency (design-by-treatment interaction model) were assessed, and no significant inconsistency was found (*P* > 0.05). Forest plot results indicated no statistically significant differences in efficacy among the various intervention types. Accordingly, the SUCRA-based ranking should be interpreted with extreme caution, as it may reflect only small and uncertain differences rather than clear superiority of any single exercise type. However, Surface Under the Cumulative Ranking Curve (SUCRA) rankings demonstrated that MT exerted the most favorable regulatory effect on MMSE scores (SUCRA = 66.7), followed by ST (SUCRA = 63.2) and RT (SUCRA = 56.1), See Figure 3 for details.

Further subgroup analyses revealed that MT interventions of approximately 60 min per session yielded more significant improvements in older adults participants' MMSE scores. Additionally, long-term exercise interventions (≥24 weeks) were associated with more pronounced effects on MMSE scores (Table 3).

**Table 3:** Subgroup analysis of the effects of different exercises on the cognitive function of the older adults.

Variable	Subgroup		*N*	SMD	95% CI	Z	Heterogeneity	
							I^2^	*P*
MMSE	Time	< 60 min	186	0.49	(0.14,0.83)	2.776	26.10%	0.245
		60–90 min	396	0.66	(−0.01, 1.34)	1.935	89.20%	0.000
		>90 min	139	0.15	(−0.45,0.76)	0.495	45.90%	0.174
	Frequency	< 3 t/w	307	0.69	(−0.20,1.58)	1.53	91.70%	0.000
		≥3 t/w	414	0.45	(−0.01,0.91)	1.924	79.10%	0.000
	Period	≤ 12 weeks	20	0.62	(−0.28,1.52)	1.353	0.00%	0.000
		>12weeks	701	0.54	(0.09,1.00)	2.336	87.50%	0.000
MoCA	Time	< 60 min	221	0.45	(0.18, 0.72)	3.305	0.00%	0.000
		60–90 min	587	1.19	(−0.29,2.68)	1.577	98.10%	0.000
		>90 min	20	0.11	(−0.76, 0.99)	0.255	0.00%	0.000
	Frequency	< 3 t/w	467	1.23	(−0.80, 3.26)	1.191	98.30%	0.000
		≥3 t/w	361	0.62	(0.10, 1.14)	2.341	75.70%	0.016
	Period	≤ 12 weeks	351	0.47	(−1.40,2.34)	0.489	96.20%	0.000
		>12 weeks	477	1.27	(0.20,2.34)	2.323	95.90%	0.000
WAIS	Time	< 60 min	60	1.01	(0.47, 1.55)	3.683	0.00%	0.000
		60–90 min	151	1.35	(0.77,1.92)	4.593	59.90%	0.083
	Frequency	≥3 t/w	211	1.26	(0.82, 1.69)	5.706	84.20%	0.108
	Period	>12weeks	211	1.26	(0.82, 1.69)	5.706	84.20%	0.108
WCST	Time	60–90 min	64	0.61	(0.11, 1.12)	2.381	0.397	0.566
	Frequency	≥3 t/w	64	0.61	(0.11, 1.12)	2.381	0.397	0.566
	Period	≤ 12 weeks	24	0.43	(−0.38, 1.24)	1.04	0.00%	0.000
		>12 weeks	40	0.73	(0.08, 1.38)	2.21	0.794	0.57

#### MoCA

A total of 7 RCTs involving 828 participants were included, with 413 in the intervention group and 415 in the control group. In the network plot, node size represented the number of studies characterizing each intervention program, while the thickness of connecting lines reflected the frequency of direct comparisons between intervention pairs.

Results showed that direct comparison studies between CON and AT were the most abundant. The inconsistency test revealed no statistically significant inconsistency (*P* = 0.47), supporting the use of a consistency model for evidence synthesis. Node-splitting analysis for closed loops (AT-RT-ST-CON) showed no significant inconsistency (*P* > 0.05). Forest plot results indicated that RT and ST exerted statistically significant effects on MoCA compared with CON. SUCRA rankings further confirmed that ST had the most favorable regulatory effect on MoCA scores (SUCRA = 94.7), followed by RT (SUCRA = 80.9).

**Figure 4:**
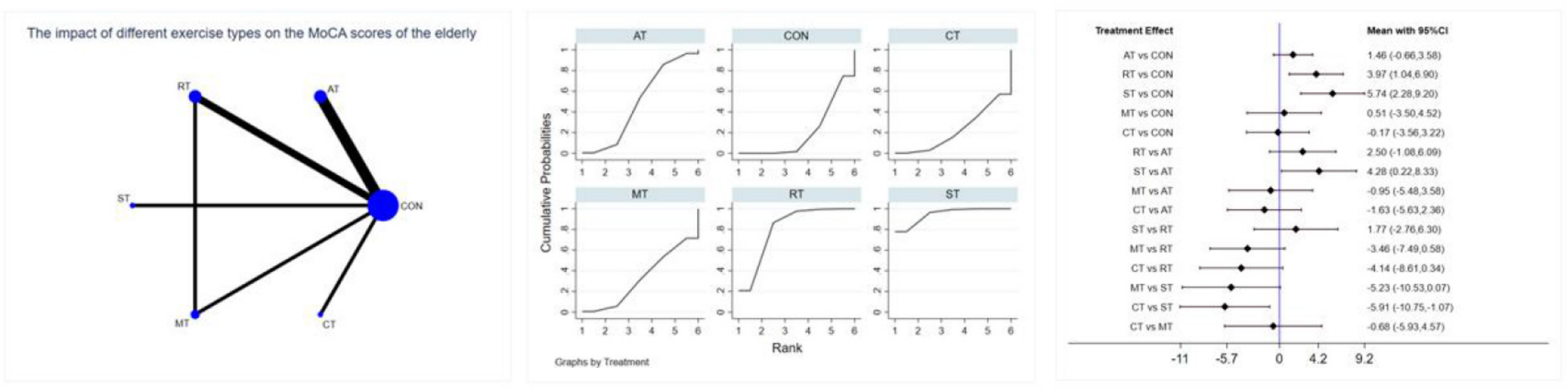
The network relationship diagram of different exercise intervention on MoCA.

Further subgroup analyses revealed that ST interventions of approximately 60 min per session yielded more significant improvements in older adults participants' MoCA scores. Additionally, medium-to-long-term exercise interventions (≥12 weeks) were associated with more pronounced effects on MoCA scores (Figure 4).

#### WAIS

A total of 4 RCTs involving 211 participants were included, with 98 in the intervention group and 113 in the control group. In the network plot, node size represented the number of studies characterizing each intervention program, while the thickness of connecting lines reflected the frequency of direct comparisons between intervention pairs.

Results showed that direct comparison studies between the CON, AT, and RT were the most abundant. The inconsistency test revealed no statistically significant inconsistency (*P* = 0.68), supporting the use of a consistency model for evidence synthesis. Node-splitting analysis for closed loops (AT-RT-ST-CON) showed no significant inconsistency (*P* > 0.05). Forest plot results indicated that AT and ST exerted statistically significant effects on WAIS compared with CON. SUCRA rankings further confirmed that ST had the optimal regulatory effect on WAIS scores (SUCRA = 98.6), followed by AT (SUCRA = 67.3).

**Figure 5:**
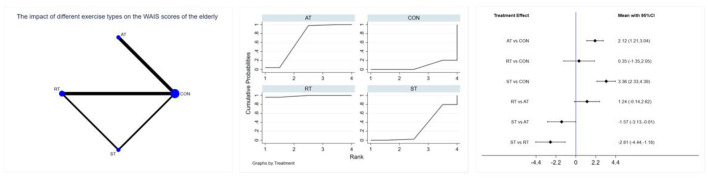
The network relationship diagram of different exercise intervention on WAIS.

Additional subgroup analyses revealed that intervention time, frequency, and duration did not modify the effect of different exercise modalities on older adults participants' WAIS scores—all exercise interventions remained statistically significant regardless of these parameters (Figure 5).

#### WCST

For the statistical analysis of WCST, a total of 2 studies were included, all of which investigated AT and reported WCST outcomes. Due to the limited number of studies (*n* = 2) and exclusive focus on a single exercise modality (AT), network Meta-analysis was not feasible, and only traditional Meta-analysis (forest plot) was conducted. These studies involved 64 older adults participants, with 35 in the intervention group and 29 in the control group.

**Figure 6:**
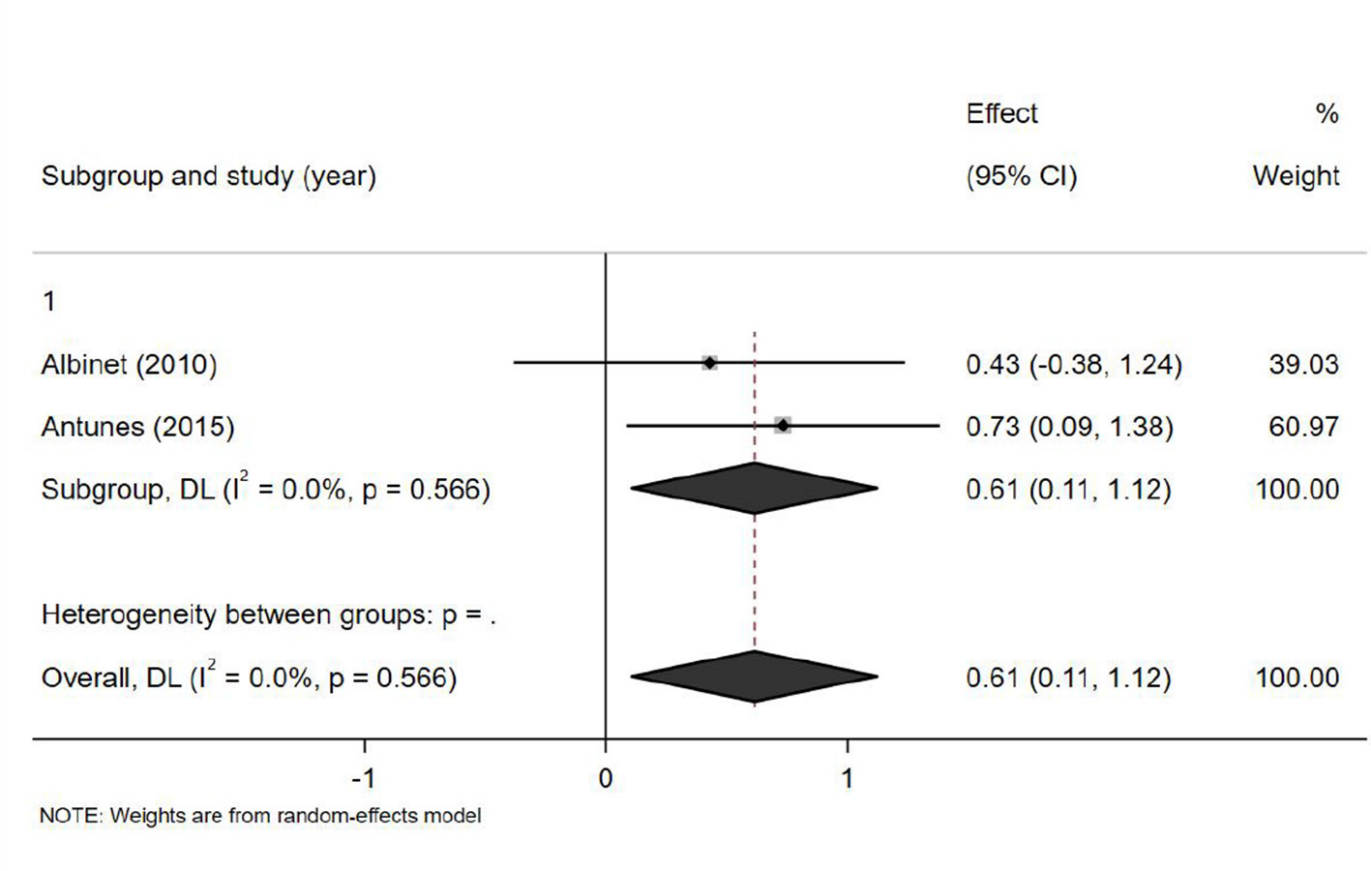
WCST's meta-analysis forest plot.

Forest plot results (shown in Figure 6) showed that WCST scores were significantly higher in the exercise group compared to the control group [standardized mean difference (SMD) = 0.61, 95% confidence interval (CI): 0.11–1.12]. Additionally, evidence from the included literature demonstrated that long-term exercise interventions—administered for ≥60 min per session, 3 times per week, and lasting ≥12 weeks—exerted a significant effect on improving WCST scores in older adults. Given the small number of included studies and limited sample size for the WCST outcome, the findings should be interpreted with caution due to the relatively low robustness of evidence.

### Publication bias

Due to the relatively small number of included studies for other outcome indicators, publication bias analysis was limited to MMSE and MoCA scales.

**Figure 7:**
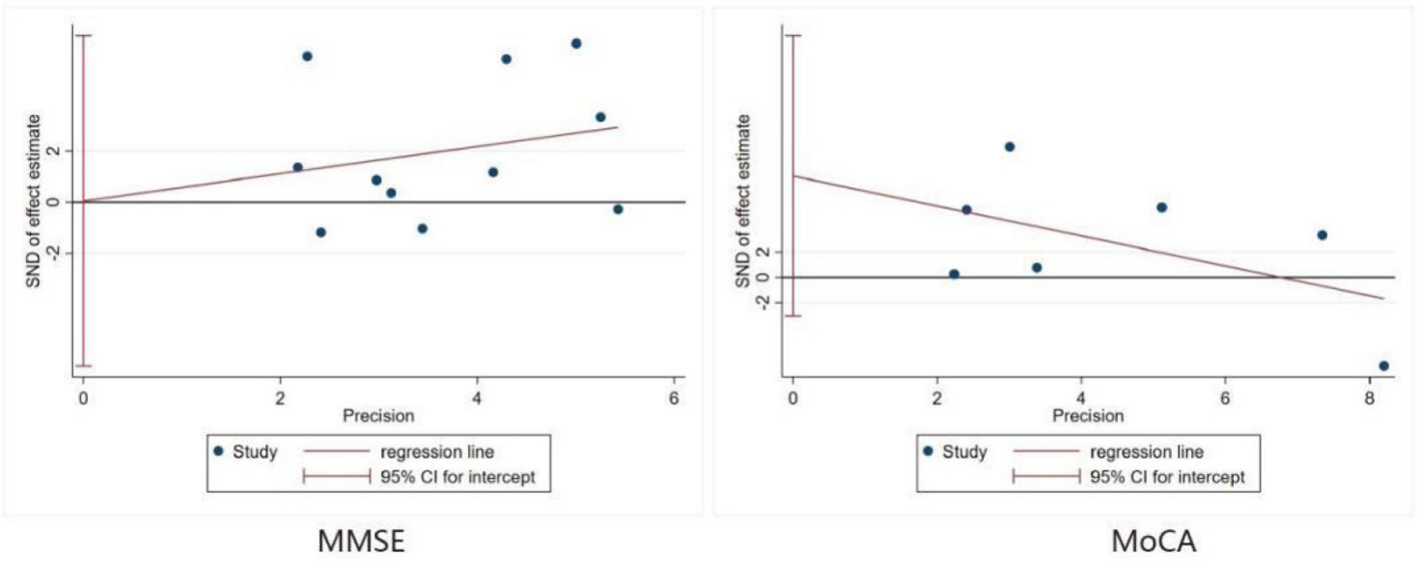
Egger plots for MMSE and MoCA.

Funnel plot results (shown in Figure 7) showed that most studies were distributed symmetrically around the mean effect size, suggesting a low risk of overall publication bias. Although a small number of data points exhibited asymmetric distribution, Egger's regression test results indicated no statistically significant publication bias for either scale: MMSE (*P* = 0.987) and MoCA (*P* = 0.122), with neither reaching the statistical significance threshold.

### Sensitivity analysis

**Figure 8:**
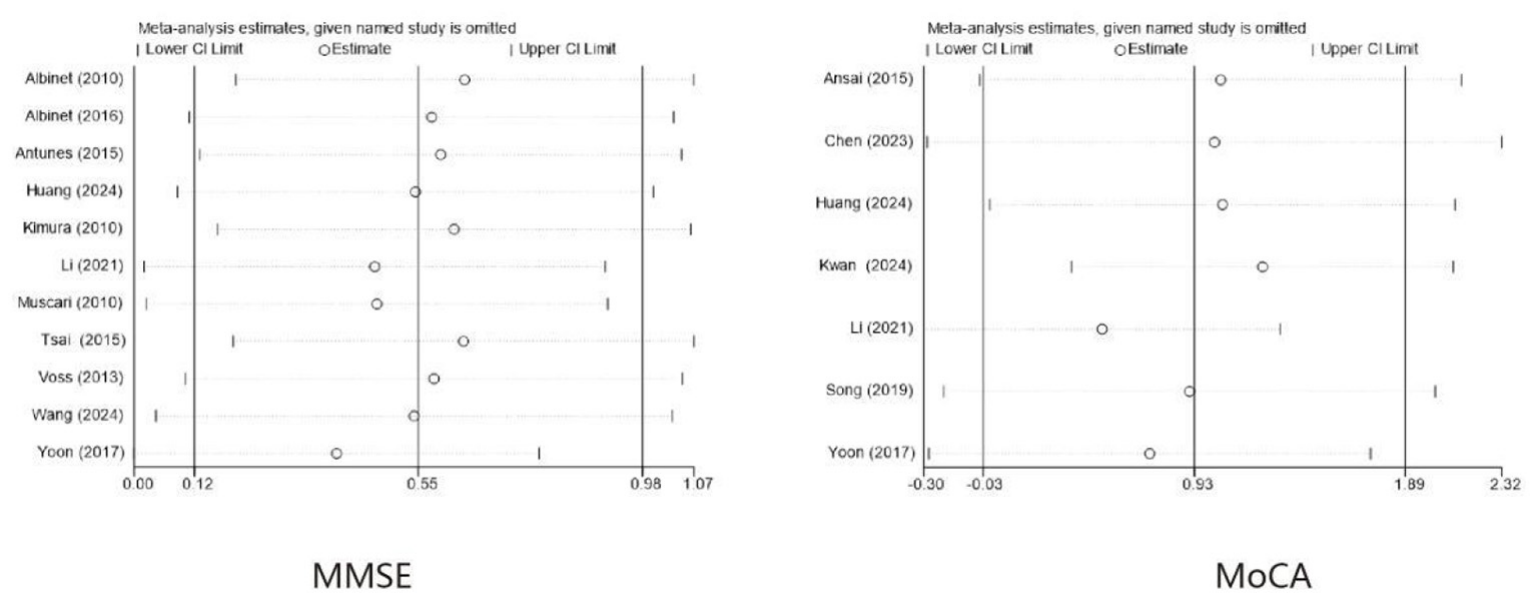
Sensitivity test chart.

To assess the robustness of the meta-analysis findings, we performed a sensitivity analysis using the leave-one-out method for outcome measures with high heterogeneity. The results indicated that sequentially excluding any single study did not substantially alter the direction or statistical significance of the pooled effect size, indicating that the findings are robust. Furthermore, although the exclusion of individual studies occasionally reduced the I^2^ value, the overall impact on the heterogeneity estimate was minimal, confirming that no single study exerted a dominant influence on the analysis (shown in Figure 8).

### Certainty of evidence

Overall, the certainty of evidence ranged from low to moderate across the assessed cognitive function outcomes, as shown in Table 4. Moderate certainty was found for the effect on cognitive function assessed by WAIS, supported by moderate heterogeneity (I^2^ = 50.6%) and adequate sample size with consistent effect direction. In contrast, most other outcomes, including MMSE, MoCA and WCST, were supported by evidence of low certainty, mainly due to serious risk of bias (e.g., inadequate blinding, incomplete reporting of allocation concealment), high heterogeneity (I^2^ = 86.1% for MMSE, 97.2% for MoCA), and imprecision (small sample size for WCST, wide 95% confidence intervals crossing the null effect for MoCA).

**Table 4:** GRADE.

Outcome	No of studies	Certainty assessment					Standardized mean effect (95% CI)	GRADE
		Risk of bias	Inconsistency	Indirectness	Imprecision	Other		
MMSE	11 RCTs	Serious	Serious	Not serious	Serious	None	0.55 [0.12, 0.98]	⊕⊕○○ LOW
MoCA	7 RCTs	Serious	Serious	Not serious	Serious	None	0.93 [−0.03, 1.89]	⊕⊕○○ LOW
WAIS	4 RCTs	Serious	Moderate	Not serious	Moderate	None	1.26 [0.82, 1.69]	⊕⊕⊕○ MODERATE
WCST	2 RCTs	Serious	Not serious	Not serious	Serious	None	0.61 [0.11, 1.12]	⊕⊕○○ LOW

## Results

This study investigated the intervention effects of different exercise modalities on cognitive function in older adults from an evidence-based medicine perspective. Results demonstrated that exercise is beneficial for improving cognitive function in older adults and can effectively delay age-related cognitive decline. Notably, distinct exercise modalities exerted differential effects: MT, RT, and ST showed greater advantages in influencing MMSE and MoCA outcomes, while their improvement effects on WAIS and WCST were not statistically significant.

These findings are partially consistent with prior related research (26, 27). For example, Lachman et al. (28) reported that RT could modulate WAIS performance and enhance memory in older adults, with more pronounced effects observed at higher resistance levels. Talar et al. (27) explored the impact of different dose parameters of AT on older adults cognitive function and found that AT performed at 50%−60% heart rate reserve (HRR) significantly improved cognitive and executive abilities. The discrepancy between these prior results and the current study's non-significant effects on WAIS/WCST may be attributed to inadequate exercise load control in the included literature. In contrast, Lachman et al. (28) stratified interventions by load and confirmed that higher resistance was associated with more significant memory improvements in older adults.

Additionally, the present study highlights that AT, RT, or multi-component training integrating both modalities may be critical for enhancing cognitive function in older adults (29, 30). Mechanistically, RT increases muscle pump activity by compressing peripheral blood vessels, thereby elevating stroke volume and cerebral perfusion, while promoting the secretion of brain-derived neurotrophic factor (BDNF) and insulin-like growth factor-1 (IGF-1) to enhance synaptic plasticity [16, 43]. Liu et al. (31) compared the effects of different RT modalities on older adults cognitive function and found that targeted AT interventions could improve cognitive function to varying degrees (7, 32, 33). Similar findings have been reported in studies of traditional exercise modalities that incorporate AT or RT components.

Notably, these mechanistic explanations are speculative interpretations based on prior literature, rather than direct evidence derived from the included trials in the present network meta-analysis.

Relevant research (34–36) has identified that alterations in carotid artery elasticity and imbalances in vascular tone (vasoconstriction-vasodilation) can exacerbate cognitive impairment. These vascular changes significantly compromise the body's capacity to deliver blood and oxygen to brain tissue, leading to the accumulation of reactive oxygen species (ROS) and subsequent neuronal damage. Exercise mitigates this process by improving cardiovascular function, increasing cerebral blood flow and oxygenation, and delivering additional nutrients to brain cells. These effects help maintain brain function, thereby delaying or reversing neurodegenerative processes and disease progression.

Notably, the high heterogeneity observed in MMSE (I^2^ = 86.1%) and MoCA (I^2^ = 97.2%) may be attributed to multiple factors, including differences in participant baseline cognitive status (cognitively healthy vs. mild cognitive impairment), comorbidities (e.g., hypertension, diabetes), exercise intensity standardization, and cognitive assessment operational procedures [6, 11, 42]. Subgroup analysis showed that 60–90 min/session, ≥3 times/week, and ≥12 weeks of moderate-intensity exercise yielded more stable improvement effects, which provides a reference for reducing heterogeneity in future studies.

Together, these findings support the cognitive benefits of exercise in older adults, but the observed modality-specific effects and corresponding rankings should be interpreted with caution. Given that these results were partly generated from indirect comparisons within the network meta-analysis and based on a limited number of trials for certain outcomes, considerable uncertainty remains regarding the precise superiority of specific exercise types.

## Limitations

Firstly, the primary outcome measures in this study included MMSE, MoCA, WAIS, and WCST. However, cognitive function is also correlated with exercise dosage and heart rate variability (HRV), in addition to exercise modality. Due to methodological inconsistencies across included studies, we were unable to systematically evaluate the independent effects of different exercise modalities on cognitive function and associated executive functions.

Secondly, the substantial heterogeneity observed in some results may be attributed to variations in cognitive assessment protocols and exercise intervention designs. Future researchers should conduct studies in accordance with standardized guidelines for measuring independent variables (e.g., exercise parameters) and dependent variables (e.g., cognitive outcomes). This standardization will reduce inter-study heterogeneity and enhance the comparability of results.

Thirdly, exercise intensity, gender, and age-related factors may moderate the effects of different exercise modalities on cognitive function in older adults. Most included studies did not report standardized exercise intensity, which prevented further subgroup analysis based on intensity and may have contributed to statistical heterogeneity. Additionally, the inherent limitations of network Meta-analysis should be noted: some exercise type pairs (e.g., ST vs. MT) had few direct comparison studies, and the small number of trials for several outcomes limits the stability of SUCRA rankings, which should be interpreted cautiously.

Fourthly, Although control conditions included routine care, health education, and usual lifestyle, all were categorized as non-exercise controls to maintain analytical consistency. Subgroup analysis by control type was not performed because the core comparison focused on exercise vs. no exercise, and further categorization would reduce statistical power and limit the generalizability of the network meta-analysis findings.

Future research should: (1) explore the efficacy of additional exercise modalities (e.g., traditional Chinese exercises, high-intensity interval training); (2) stratify analyses by gender, age groups, and gender-specific age subgroups; and (3) further elucidate the moderating roles of these factors to better understand the targeted effects of exercise on older adults cognitive function.

## Conclusion

This study performed a network meta-analysis to systematically evaluate the intervention effects of various exercise modalities—including AT, RT, MT, CT, and ST—on cognitive function in cognitively healthy older adults and individuals with MCI. Results confirmed that physical exercise effectively improves multi-dimensional cognitive function in older adults, with intervention advantages of different exercise modalities exhibiting significant dimension specificity.

This study provides high-level evidence-based medical support for promoting cognitive health in older adults. In clinical practice and public health initiatives, individualized exercise prescriptions can be developed based on older adults individuals' cognitive assessment results and health status: prioritize MT for global cognitive function maintenance, select ST or RT for fine cognitive function and intelligence enhancement, and emphasize AT for executive function improvement. Future research should adopt standardized exercise protocols and assessment procedures, expand sample sizes, and incorporate confounding factors (e.g., gender, age stratification, comorbidities) to further explore the cognitive benefits of traditional exercise modalities, thereby providing more comprehensive scientific evidence for maintaining older adults cognitive function.

## Data Availability

The original contributions presented in the study are included in the article/supplementary material, further inquiries can be directed to the corresponding author.
